# Designing and evaluating provider results-based financing for tuberculosis care in Georgia: a realist evaluation protocol

**DOI:** 10.1136/bmjopen-2019-030257

**Published:** 2019-04-14

**Authors:** Bruno Marchal, Ariadna Nebot Giralt, Lela Sulaberidze, Ivdity Chikovani, Ibukun-Oluwa Omolade Abejirinde

**Affiliations:** 1 Department of Public Health, Institute of Tropical Medicine, Health Systems and Equity Unit, Antwerp, Belgium; 2 Curatio International Foundation, Tbilisi, Georgia

**Keywords:** realist evaluation, research protocol, georgia, results-based financing, health policy

## Abstract

**Introduction:**

In 2016, Georgian researchers and policymakers were developing a policy to improve the performance of the national tuberculosis (TB) control programme. The research programme ‘Designing and Evaluating Provider Results-Based Financing for Tuberculosis Care in Georgia: Understanding Costs, Mechanisms of Effect and Impact (Results4TB)’ was initiated to inform the policy formulation phase, document the policy implementation and assess the effectiveness, cost-effectiveness and the processes of change. To achieve this, the research team intends to combine an impact evaluation, a cost-effectiveness study and a realist evaluation (RE) within an overarching theory-informed design. This protocol is the RE component of the programme.

**Methods:**

A realist methodological approach will be adopted to guide the research design and evaluation. RE answers the question of ‘what works in which conditions for whom?’ and starts with the development of an initial programme theory (IPT). The IPT will feed into other phases of the realist research cycle (study design, data collection, data analysis and synthesis and theory refinement). Data will be collected in a multiple embedded case study design (five intervention and three control sites) through document reviews, in-depth interviews, non-participant observations and context mapping at facility and national levels. Additional data from other research components (cost-effectiveness and impact evaluation) will aid data triangulation.

**Ethics and dissemination:**

The Institutional Review Boards of the National Centre for Disease Control and Public Health in Georgia (ref. IRB # 2018–019) and the Institute of Tropical Medicine, Antwerp (ref. IRB #- 1240/18) have granted ethical approval to the study.

**Trial registration number:**

ISRCTN14667607

**Strengths and limitations of this study**

- This study uses an integrated theory-informed design for a combined impact, cost-effectiveness and process evaluation of a policy, thus addressing a currently debated methodological challenge.
- In addressing a policy that is being developed, the study may be faced with unexpected turns in the policymaking and implementation phases.
- The realist evaluation cycle fosters a flexible and iterative structure for learning and reflection. For example, applying a realist approach led to the formulation of a more comprehensive and contextually relevant policy package.

## Introduction

Under the national tuberculosis (TB) programme in Georgia, Eastern Europe, TB service is provided to patients free of charge. At outpatient level, services are provided at TB units by TB doctors and TB nurses. There are currently 68 TB units in the country: 58 semiurban (located in district centres) and 10 urban TB units.

Recently, semiurban TB units were administratively integrated into district and regional level primary healthcare (PHC) centres, most of which are private. Therefore, only a few TB units remain as separate public institutions, existing mainly in the capital and other major cities of the country (10 urban centres).

TB service integration into PHC was part of a wider healthcare privatisation process whereby although it was not profitable from a private sector perspective, the government (represented by the Ministry of Health through the national TB programme) and private sector providers agreed that the latter will provide TB services. This agreement was scheduled to expire in 2018.

While Georgia has made substantial progress in managing TB, challenges still remain. Data from 2017 indicate high rates of drug-resistant TB (DR-TB) in the Georgian population. The incidence was 11% in new TB patients and 30% in those previously treated.^1^ In addition, there is suboptimal adherence to treatment among DR-TB patients in the country—every fourth DR-TB patient quits treatment prematurely. Poor coordination between PHC and TB units leading to fragmented TB care was assumed to contribute to poor treatment adherence and loss to follow-up (LFU).^2 3^

During discussions on policy options to improve TB service provision in the country, results-based financing (RBF) of providers was proposed as a viable approach to motivate healthcare providers not only to continue providing TB care but also to improve treatment adherence in TB patients. Policymakers’ willingness to pilot the RBF model for TB initially materialised as a standard concept note to the Global Fund in 2015, where in addition to other programmatic needs, the government requested financial support from the Global Fund for technical assistance to design and implement the RBF model.

In 2016, Georgian and international researchers drafted a proposal aiming to support the Georgian government in developing a policy to improve the performance of the national TB control programme and generate evidence on the proposed policy. Consequently, a research programme ‘Designing and Evaluating Provider Results-Based Financing for Tuberculosis Care in Georgia: Understanding Costs, Mechanisms of Effect and Impact (Results4TB)’ was initiated. Results4TB aims at informing the policy formulation phase, documenting the implementation of the policy and assessing the effectiveness, cost-effectiveness and the processes of change. To this end, the research team is combining an impact evaluation of the policy trial with a cost-effectiveness study and a realist evaluation (RE). Essentially, instead of developing three separate substudies (impact, cost-effectiveness and process evaluation), each addressing a different set of research questions, we will develop a theory-informed design that integrates all three components. Trial designs often include implementation fidelity evaluations, process evaluations or context mapping,^4^ and there have been calls for informing such evaluations with theory.^5 6^ The Results4TB study therefore goes a step further: not just the process evaluation, but also the overall study design will be theory informed.

This paper presents the protocol of the RE component, spelling out the objectives, research questions, methods and ethical considerations.

### Objectives

The objective of the RE component is to identify the mechanisms of change and the contextual factors that enhance or undermine the effectiveness of the provider RBF policy, defined in terms of adherence and treatment outcomes.

The research questions include (1) How is the performance based financing policy designed and by whom? (2) How is the policy implemented in the study sites? (3) How do the actors respond to the policy? (4) What are the contextual conditions needed for the policy to work? (5) What are the underlying mechanisms that explain how the policy contributes to changes in the practice of TB service providers? (6) What are the underlying mechanisms that explain how the policy contributes to a change in treatment adherence?

## Methods

### The methodological approach

Pawson and Tilley developed the RE approach arguing that in order to be useful for decision makers, rather than merely addressing the question of ‘does it work?’, evaluations need to answer the question ‘what works in which conditions for whom?’.^7^ In order to meet this need, realist evaluators therefore aim to identify the underlying generative mechanisms that explain how an intervention leads to its outcomes and in which context this occurs. Based on critical realism, RE considers that interventions work (or not) because actors respond to what is provided by the intervention (or not). The interaction between ‘intervention’ and ‘actors’ in specific ‘contexts’ therefore triggers mechanisms that lead to outcomes.

**Figure 1:**
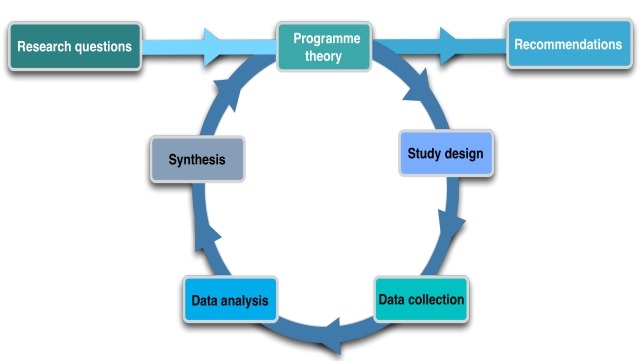
The realist cycle.

REs start with an initial programme theory (IPT), on the basis of which the study design is based. The IPT explains how a programme is expected to generate outcomes by showing which mechanisms will be triggered among different groups of actors. It also identifies the necessary contextual conditions needed for the programme, in our case the Results4TB policy, to work. In other words, the IPT is a hypothesis that will be tested subsequently and iteratively through empirical studies. We structured our protocol following the steps of the realist cycle (figure 1).^8^ The IPT, which has been formulated during stakeholder workshops, is briefly presented, following which additional components of the Results4TB RE are outlined as part of the research protocol.

### Step 1: Developing the IPT

**Figure 2:**
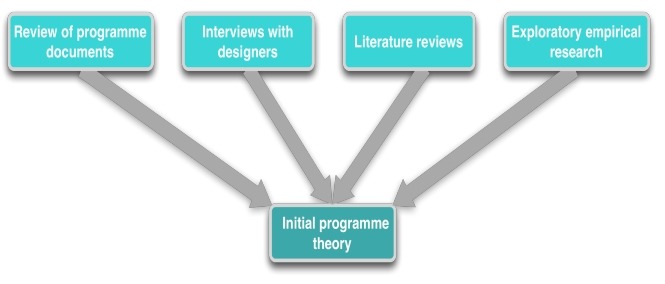
Eliciting the programme theory.

Several data sources can be used to elicit the IPT (figure 2). These include programme documents including policy briefs, concept notes and logical frameworks. A second source is interviews with the designers, funders and/or implementers of the policy, combined with on-site observations. Third, past experience, findings of previous evaluations or research studies are reviewed. Finally, in some cases, exploratory research may be carried out.

In this project, we combined existing literature reviews, workshops with policymakers and key stakeholders involved in TB care. Furthermore, discussions within the research team were instrumental in refining and constructing the IPT. First, we built on a review of the literature on adherence carried out in the frame of the RELIVING study^9^ and on literature reviews carried out on performance-based financing (PBF) and RBF, including one on PBF in low-income and lower middle-income countries.^10^

Second, we used the unique opportunity to involve both researchers and policymakers at the initial phase of the project by organising two workshops. During the workshops, we used participatory and interactive techniques to obtain a clearer insight on the proposed RBF policy and why the designers, implementers and other stakeholders think the policy may or may not work. Workshop participants ranged from TB providers to Ministry of Labour, Health and Social Affairs (MoLHSA) policymakers and representatives of the Global Fund. We used cause mapping and concept mapping^11^ to clarify how the participants understood the problem (first workshop) and the potential policy options, discussing why one option would be more effective than others (second workshop). The results were discussed by the research team members and framed on the background of findings from the literature review.

The result of the above process was that we ended up with a more complex policy; it became evident that the RBF for TB providers required expansion to address other challenges in the service delivery pathway. Consequently, in response to the input from all stakeholders, the policy was defined as a package of interventions, including

- Incentive payments (bonuses) to TB teams based on performance indicator (ie, TB patient retained on treatment).
- Trainings for all members of a TB team (a TB doctor, a family doctor, a directly observed therapy (DOT) nurse, a rural nurse) on principles of integrated and patient-centred care, and on managing TB treatment side effects and comorbidities (for family doctors and TB doctors).
- New roles and responsibilities were ascribed to TB team members which were better aligned to the scope of their professional competencies and in order to ensure integrated patient centred approach for TB.
- Launch of new treatment and monitoring tools such as facility managers guidelines on implementing the policy, case management plan for patients, instruments for monitoring integrated team performance, instruments for verification of performance indicators and the incentive scheme.

**Figure 3:**
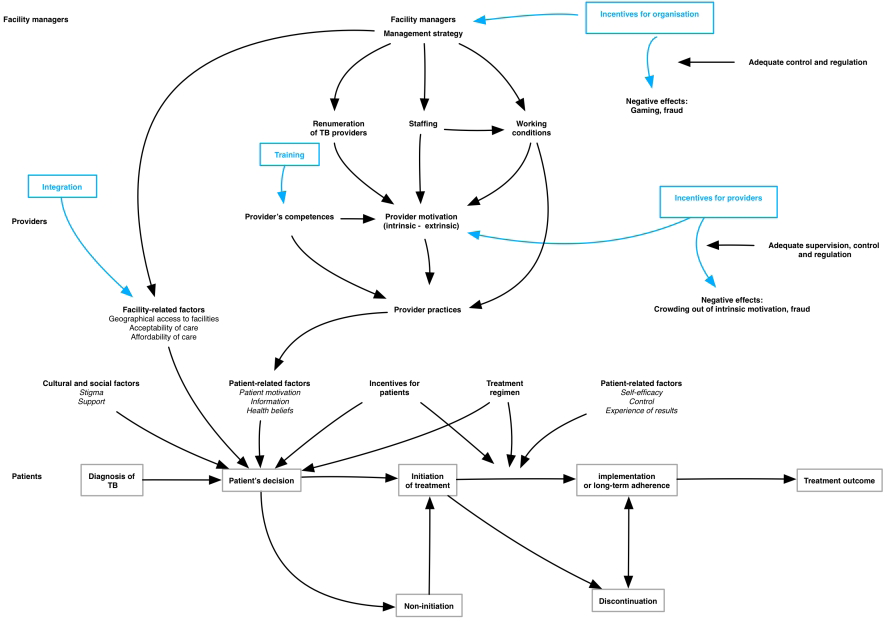
Initial programme theory. TB, tuberculosis.

This process led us to formulate the IPT as follows (figure 3):

- Financial incentives will stimulate facility managers to (1) continue providing TB services, (2) remit incentives to TB providers within their facilities and (3) optimise their TB service activities (ie, *mechanism of organisational self-interest*), on the condition that incentives are considered to be adequate and that sufficient monitoring and regulation systems are in place, in the absence of which gaming and fraud could occur.
- Provider incentives will increase their extrinsic motivation (*mechanism*) if the staffing and working conditions are adequate. Motivated and competent providers will provide better adherence support to patients, if supervision, monitoring and regulation are adequate. If not, crowding out of intrinsic motivation and gaming may occur.
- Training for providers (eg, on side effect of drugs, management of comorbidities and patient-centred care) will enhance their competence, which leads to higher self-efficacy. The latter, combined with an increase in extrinsic motivation, contributes to behaviour change; enabling patients to better adhere to the treatment.
- TB patients who are informed, motivated and supported by providers, and trained to develop the needed skills, will initiate adherence if the social and cultural context is favourable (eg, no stigma), if the facility is accessible, acceptable and affordable and if they consider the treatment regime as acceptable.
- The integration of TB care will improve the continuum of care between general and specialised TB services and thus facilitate the patient to correctly follow the treatment and care pathway towards being cured.
- TB patients who experience positive effects, feel capable of managing their health condition (ie, mechanism of self-efficacy) and feel in control are more likely to maintain long-term adherence, on condition that they consider the treatment regime as acceptable.

The formulated IPT will inform the study design and other phases of the realist cycle.

### Step 2: Study design

Given the research questions, we will adopt the multiple embedded case study design.^12^ This design is often used in realist research in health^8^ and has the advantage that it is well adapted to dynamic concepts or interventions, such as a policy in the formulation or implementation phase.^13^

We define the case as the uptake and implementation, by facility managers and service providers, of the policy that will be introduced by the MoLHSA to improve TB drug adherence and treatment outcomes. The unit of analysis is the facility, which includes first-line and second-line health facilities in which TB care is provided. Within each facility, we focus on the influence of the policy on the interaction between managers, service providers and patients, and on TB service delivery. In RE, the selection of the study sites is purposive: ideally, sites should enable ‘testing’ of the IPT in all its dimensions. The trial of the policy will be implemented in eight intervention and eight control facilities. For the RE, five intervention sites and three control sites will be chosen for data collection. Site selection will be purposive, based on the following criteria:

- Facilities of different types will be selected using ownership status (private-for-profit vs public) and organisational structure (large chain vs independent facility).
- Different subsets of service delivery modality-specialised TB services versus TB units integrated in PHC centres.
- Location—semiurban versus urban.

### Step 3: Data collection

We will use a range of data collection techniques: document reviews, in-depth interviews, non-participant observations and a context mapping tool (table 1). Additional data from other components of Results4TB will also be used to triangulate the data. Where possible data collection tools will be tested during a short pilot phase of the policy prior to full trial.

**Table 1:** Overview of data collection methods and targeted minimum numbers

Tools	Data source	Content	Minimum numbers	Time period	Responsible for data collection	Data collection technique
IDI-providers	Service providers: TB doctor, TB nurse, family doctor, rural doctor, rural nurse	Personnel perspectives	40 (5 interviews/site)	One year after the intervention start	Researchers	Face-to-face interview (IDI guides)
IDI-facility managers	Facility manager, clinical manager	Managers perspectives	16 (2 interviews/site)	One year after the intervention start	Researchers	Face-to-face interview (IDI guides)
IDI-TB coordinator	TB coordinator	TB coordinators views	5	One year after the intervention start	Researchers	Face-to-face interview (IDI guides)
IDI-patients	Patient	Patients perspectives	48 (3 DS+3 DR patients per site)	One year after the intervention start	Researchers	Face-to-face interview (IDI guides)
IDI-national level respondents	MoLHSA, National Centre for Disease Control, the Global Fund, TB programme, Social Service Agency	National-level key actors’ perspectives	5 interviews	15 months after the intervention start	Researchers	Face-to-face interview (IDI guides)
IDI-network HQ	Top managers from each network	Network specific views	3 interviews	One year after the intervention start	Researchers	Face-to-face interview (IDI guides)
Non-participant observation of consultations	TB unit	TB consultations	24 (2 DS patient+1 DR patient per facility) (Patients who are interviewed)	One year after the intervention start	Researchers	Observation
Non-participant observation of integrated team meetings	Facility	Integrated team meetings	15 (3 per facility in intervention sites only)	1 observation per site at beginning, mid-term, and near end	Researchers	Observation
Local context mapping tool	Managers	Conditions for intervention implementation		First 2 months and 1 year after the intervention start	Researchers	Face-to-face interview (IDI guide)+informal observations
National context mapping tool	Policy makers	Conditions for intervention implementation		Continuous	Researchers	Face-to-face interview (IDI guide)+informal observations+meetings with in the CIF team

DR, drug sensitive; DS, drug resistant; HQ, headquarters; IDI, in-depth interview; MoLHSA, Ministry of Labour, Health and Social Affairs; TB, tuberculosis.

#### Document review

We will carry out a document review to collect data for research question 2 (ie, how the policy is implemented in the study sites). The focus will be on finding evidence of the initiation and process of policy implementation at the facility. Facility reports, TB programme reports, data collected during policy implementation, supervision and verification reports, and other relevant policy-related documents will be used. At study sites, documents related to monitoring of TB clinic activities, such as activity reports, will be screened for information on implementation process and problems. Where possible, electronic versions will be collected and entered in NVIVO software. If not, paper versions will be collected.

#### In-depth interviews

In order to address research questions 3–6, we will carry out in-depth interviews with different actors in the policy trail: facility managers, TB service providers, TB coordinators (who will conduct policy supervision and verification role), TB patients and national-level respondents. Interview guides will be developed in collaboration with the other project teams and with the local partner in Georgia. The guides will be first translated into Georgian, piloted and modified if needed. Table 1 presents the estimated number of interviews per respondent type and level.

The following procedure will be used:

- All potential respondents will be invited to participate in the study by personal invitation using a snowball approach. The researchers will invite facility managers, healthcare personnel and national-level respondents. TB doctors will extend invitations to their patients, after receiving training from the research team on recruitment and ethical procedures.
- Interested potential respondents will then receive and be guided through a participant information sheet, explaining the study objectives and procedures.
- If participants are still interested to be interviewed, an interview will be arranged according to their preferred time and location.
- At the start of the interview, the researchers will provide detailed information about the study and the interview and answer any question.
- The informed consent form will be presented and explained by the researcher. Informed consent will be sought at the start of the interview, as well as permission to record the interview.
- All recorded transcripts will be transcribed verbatim. A sample of interviews will be translated to be used in an initial coding training workshop and will also allow for quality assessment by the non-Georgian team members.
- Field notes and memos will be entered in an electronic form.
- All transcripts and related memos will be entered in NVIVO for subsequent data analysis.

#### Non-participant observation

To collect information related to research questions 2–6, in addition to individual interviews, we will conduct non-participant observations at TB clinics and observe the integrated TB team meetings, using observation guides. Where possible, we will aim to observe the clinical encounter of each patient who has agreed to be interviewed. Researchers will ask for written informed consent before conducting any observation. Patients will be able to opt out of observations, yet still participate in an interview.

#### Context mapping tool

In order to document the context of each facility and to identify contextual conditions for the intervention to work (research question 3), we will develop and use a facility context-mapping tool. This will be used to document key facility-related issues, including organisational structure, decision spaces, flow of funds and information, and the TB patient pathway. A national context mapping tool will be developed and used to identify the key stakeholders, track their engagement with the policy over time, identify key political and policy events and other events that may influence the policy implementation and or its outcomes.

#### Data from the other Results4TB study components

Any RE starts from the observed outcomes, working backwards to identify the mechanisms, actors and contextual factors that explain them. To achieve this, in addition to the data collected under the RE, we will draw from the data collected by other parts of the trial. For example, data related to effectiveness that we could use could include inpatient and outpatient treatment initiation rates; treatment adherence rate (disaggregated into ‘completed’ and ‘loss to follow-up’); treatment outcome rate (disaggregated into ‘cured’, ‘failure’, ‘died’ and ‘not evaluated’); comorbidities rate; rate of referral to other outpatient facilities and rate of hospitalisation for management of comorbidities.

### Step 4: Data analysis

RE is method neutral. Data analysis methods should follow the best practices of the disciplines of which it borrows the methods used. In general, the analysis aims to develop intervention-context-actors-mechanism-outcome configurations which serve as an analytical heuristic.^14^

During the first round of analysis, the following guiding questions will be used: What are the observed outcomes? What is the actual implemented intervention? How was it carried out (duration, intensity, process)? Who delivered the intervention or who are the actors involved? How did the intervention reach the target population and to which degree (coverage)? Can the observed results be linked to the actual intervention?

In a second round of analysis, we will aim to assess the contribution of the actual intervention to the observed outcomes. Guiding questions include How can the link between the actual intervention and the actual outcomes be explained (mechanisms)? Which context conditions facilitated the policy package to work (or not)? Which conditions constrained the policy? Are there alternative explanations for the observed outcomes (ie, other interventions or events that may have contributed to the observed outcomes)?

In line with realist principles, a thematic coding approach will be used based on the core elements of the IPT. Framework analysis^15^ will be used because it allows for the inclusion of both a priori and emergent concepts. In the first round of analysis, data will be categorised using the intervention-actor-context-mechanism-outcome configuration. New interpretations will emerge in subsequent rounds of coding, leading to a refined analysis. This results in descriptions of the actual intervention, its effects (both intended and unintended, positive and negative), the contextual elements and the underlying mechanisms generated in actors.

### Step 5: Synthesis

We will use methodological triangulation by combining different qualitative data collection techniques and quantitative analysis, drawing on data from different sources (interviews, observations, document reviews and the other Results4TB components). The findings of each site will be summarised in reports. Subsequently, a comparison of the facilities will be carried out. The end result will be refined programme theories that specify how the policy played out in different types of contexts and how it affected the outcomes of adherence and LFU, the endpoints of the trial study.

#### Documentation of the research process

The research field team members will write field notes and keep a research diary in the form of a qualitative log, that also tracks the different sources of data collection. Contact summary sheets will be written after each interview or observation to record the researchers’ impressions and emerging ideas, and allow new insights to be documented for later retrieval. During the analysis, analytical memos will also be written to allow for an iterative approach. Case analysis meetings will be held regularly in the form of feedback and discussion meetings by the research team. These should allow critical review of observations and preliminary findings and conclusions, as well as peer review.

## Ethics and dissemination

We will carry out this study according to the principles stated in the Declaration of Helsinki as amended in 2013.^16^ Informed consent will be secured from all respondents using information sheets and written informed consent forms, which will be translated to Georgian language. Findings will be published in an open-access peer-reviewed journal and further disseminated at international conferences.

## Patient and public involvement

Patients will not be invited to comment on the study design nor to develop patient relevant outcomes or interpret the results. Policy makers, health workers and representatives of the Global Fund were, however, involved in codesigning the initial programme theories during participatory stakeholder workshops. They may be involved in data validation workshops to refine the tested programme theories.

## Discussion

This RE is part of a large theory-driven study that combines a policy trial, cost-effectiveness and RE studies. The approach offers flexibility and allows iterative reflection while combining different research paradigms. The aim is not to force one paradigm on the other but rather to jointly inform all study components with theoretical insights. This will better integrate data collection and analysis and lead to an integrated assessment of the policy. In this way, the study addresses a hotly debated issue in circles of trials and RE.^17–20^

## Supplementary Material

Reviewer comments

Author's manuscript
