# Supplementary material for: Designing and evaluating provider results-based financing for tuberculosis care in Georgia: a realist evaluation protocol
Source: BMJ Open. 2019 Apr 14;9(4):e030257. doi: 10.1136/bmjopen-2019-030257 (PMC6500265; doi:10.1136/bmjopen-2019-030257)
Supplement: Reviewer comments [file bmjopen-2019-030257.reviewer_comments.pdf]

PEER REVIEW HISTORY

BMJ Open publishes all reviews undertaken for accepted manuscripts. Reviewers are asked to complete a checklist review form (**http://bmjopen.bmj.com/site/about/resources/checklist.pdf**) and are provided with free text boxes to elaborate on their assessment. These free text comments are reproduced below.

ARTICLE DETAILS

**TITLE (PROVISIONAL)**	DESIGNING AND EVALUATING PROVIDER RESULTS-BASED FINANCING FOR TUBERCULOSIS CARE IN GEORGIA: A REALIST EVALUATION PROTOCOL
**AUTHORS**	Marchal, Bruno; Giralt, Ariadna; Sulaberidze, Lela; Chikovani, Ivdity; Abejirinde, Ibukun-Oluwa

This article was not externally reviewed at BMJ Open. Protocols that have been independently assessed prior to submission to BMJ Open are usually fast-tracked to publication on the grounds that further substantial changes will not be possible. This independent assessment will usually be external, independent review for both a substantial grant award from a non-commercial or government funder and ethics approval.
